# Forecasting renewable energy for environmental resilience through computational intelligence

**DOI:** 10.1371/journal.pone.0256381

**Published:** 2021-08-20

**Authors:** Mansoor Khan, Essam A. Al-Ammar, Muhammad Rashid Naeem, Wonsuk Ko, Hyeong-Jin Choi, Hyun-Koo Kang

**Affiliations:** 1 School of Electronics and Materials Engineering, Leshan Normal University, Leshan, China; 2 Department of Electrical Engineering, College of Engineering, King Saud University, Riyadh, Saudi Arabia; 3 School of Artificial Intelligence, Leshan Normal University, Leshan, China; 4 GS E&C Institute, GS E&C Corp., Jongno-gu, Seoul, South Korea; 5 Department of Electrical and Electronic Engineering, Hannam University, Daedeok-gu, Daejeon, South Korea; Ton Duc Thang University, VIETNAM

## Abstract

Wind power forecasting plays a key role in the design and maintenance of wind power generation which can directly help to enhance environment resilience. Offshore wind power forecasting has become more challenging due to their operation in a harsh and multi-faceted environment. In this paper, the data generated from offshore wind turbines are used for power forecasting purposes. First, fragmented data is filtered and Deep Auto-Encoding is used to select high dimensional features. Second, a mixture of the CNN and LSTM models is used to train prominent wind features and further improve forecasting accuracy. Finally, the CNN-LSTM deep learning hybrid model is fine-tuned with various parameters for reliable forecasting of wind energy on three different offshore Windfarms. A state-of-the-art comparison against existing models is presented based on root mean square error (RMSE) and mean absolute error (MAE) respectively. The forecasting analyses indicate that the proposed CNN-LSTM strategy is quite successful for offshore wind turbines by retaining the lowest RMSE and MAE along with high forecasting accuracy. The experimental findings will be helpful to design environment resilient energy transition pathways.

## Introduction

Renewable energy has been studied for a long time due to high electricity generation costs and global warming. Sustainable energy production plays a vital role in reducing global warming and the impact of climate change. Therefore, the energy sector is now more focused on seeking the appropriate tools for the widespread use of renewable energy resources. Global demand for renewable energy rises to 30 percent relative to 435 installed capacities, with an annual growth of 17 percent in recent years. In 2020, renewable energy provided an estimated 12% of total energy output worldwide [1]. Wind power consumption has increased nearly twice, while biogas consumption has increased by 2.8% over the past five years. As a result, overall greenhouse gas emissions are decreased by 3% and environmental damage by 23%, respectively. Estonia has taken steps to expand the use of renewable energy in the form of the Short Assessment of Alternative Energy Sources (AES) by 30%, 45%, and 80% between 2020, 2030, and 2050 [2].

Renewable wind power is further categorized into short-term and long-term wind power production. Short-term wind power varies from few minutes to a day, while long-term wind power is based on weeks, months, or even years. Both short-term and long-term wind data can be utilized to forecast wind power generation. However, short-term wind power forecasting is deemed preferable compared to long-term power forecasting. Power sectors of different countries use various wind power forecasting methods to generate sufficient wind energy. These methods largely focused on either mathematical or physical models, whereas the hybrid models rely on the integration of both models is also popular in the energy sector. Physical models can be affected by particular characteristics such as wind turbine position, barriers, surface coarseness, and blade turbulence, etc. Physical models are specifically used for long-term wind power forecasting [3]. Contrary, the mathematical models use historical wind data to estimate potential power generation [4,5] and may also take advantage of various hybrid frameworks such as machine learning and deep learning-based power forecasting. Common forecasting methods include Support Vector Regressor (SVR) [6], Multi-layer perceptron [7], deep neural network [8], as well as a combination of various related methods [9,10].

Wind power has uncertain characteristics and weak controllability, which also raises the problem of inconstancy and fluctuations in power systems. Apart from that, air velocity may also be influenced by the possibility of hazardous modes and postures. Therefore, an effective power monitoring system is needed for efficient transmission and to resolve the power generation demands. Various estimation strategies have been introduced and structured to examine and estimate sustainable wind power [11]. Wind power forecasting strategies are generally classified into three theoretical classifications, including Forecast Analysis (NWP), Survey Data Analysis (SDA), and a mixture of the two approaches [12,13].

Probabilistic units are typically known to estimate the relevant NWP since these approaches have become more and more significant at periodic intervals with better accuracy. However, it would be very difficult to develop an efficient numerical framework without any in-depth analysis of the systems engineering as well as the wind area atmosphere. These types of models need to construct certain variables with the aid of illustrative variables, census data algorithms, to overcome the association between empirical evaluation and extracted wind properties. These methods may only need observational data for estimates and are also fairly convincing to widespread forms of research implementation. Despite their significance, the accuracy of these models has been declining over time. Nowadays, most of the statistical techniques are used in collaboration with the Artificial Neural Network (ANN) [14], Convolution Neural Network (CNN) [15], Support Vector Regression (SVR) [16], and Back Propagation Neural Network (BPNN) [17] respectively. Several data science algorithms have been developed for complex database systems to efficiently use and train data to solve forecasting problems in multiple domains. Such algorithms use specific behavior of data and afterward generates predictive outcomes. CNN is a supervised learning algorithm that uses a perceptron to extract estimations from adaptive learning. Alternatively, BPNN is also a supervised learning algorithm that determines the cumulative marginal distribution of feed-forward backpropagation in a fully connected neural network.

The wind turbine data is based on a time series with a real-time wind speed reading for a particular area. Wind power forecasting models mainly use these readings along with other characteristics to predict potential power output for that area. For short-term forecasting, minutes and hourly time series data are more accurate as they can be used for stochastic wind signals and have been used in many models such as Kalman filters [17], Box-Jenkins [18], as well as ANN [19]. The Box-Jenkins model uses probabilistic approximation and cannot be used to predict future wind power. Therefore, their model involves a full assessment of the distinct forecasts. The Kalman filter is considered to be a direct symmetric estimator, as it uses a limited direct estimation feature relative to Box-Jenkins and thus requires less computational costs. As a result, Kalman filters are steady and needless computation, but Box-Jenkins provides more effective results with a significant number of simulations. However, considering the risk of diverse knowledge and extremely nonlinear wind behavior, the ANN has gained more momentum in handling complex and dynamic information. ANN estimates forecasting based on neural structures such as multilayer perception with formal specifications that may not be fully suitable for wind power forecasting. The predictive potential of these approaches falls beyond the longer forecast horizons [18,20,21]. As mentioned, our proposed deep learning model based on combined approach of CNN and LSTM which has a significant advantage over certain state-of-the-art approaches.

The main contribution of this paper is as follows:

- First, we use the NREL toolkit to analyze and extract offshore wind power data. The collected data is further divided into three regions that span large-scale offshore wind turbines in the United States.
- Second, wind power data is primarily based on time series, and LSTM is well-suited to handle time series forecasting regardless of duration lags. Thus, a combined approach of CNN and LSTM is used to analyze hidden features of offshore winds for reliable wind power forecasting.
- Next, data is preprocessed using deep auto-encoders prior to wind power forecasting to minimize the error ratio. Offshore winds are diverse and continuously changing, whereas deep auto-encoders can learn low-dimensional data efficiently and with minimal reconstruction error.
- Furthermore, the proposed CNN-LSTM forecasting model is optimized using fine-tuned parameters for each offshore region, and the performance is compared to the current state-of-the-art methods.
- As per empirical analysis, the proposed method demonstrated excellent forecasting accuracy with a low error ratio at various intervals, making it more suited approach for offshore wind power forecasting.

## Literature review

Current wind power forecasting technologies have limitations and needed to be enhanced to estimate actual wind power generation. It is essential to investigate the different perspectives of wind power to design an effective model. Most recent researches have been focused on hybrid techniques to take advantage of integrated methods. Individual approaches usually produce low efficiency than hybrid approaches, while the hybrid approaches can be further divided into key parts [22]. A hybrid approach combined with computational intelligence can be more effective and widely studied in energy and power forecasting. For instance, Kaluri et al [23] used predictive power of rough sets to forecast battery power life and, Maddikunta el al [24] used hybrid algorithmic strategy for efficient power communication between networks. A hybrid computational intelligence technique can learn effective model features from wind direction and wind speed adjacent to wind turbines before actual wind power forecasting [25]. The outcomes of hybrid deep learning approaches recommended that the multiple neural networks must efficiently enhance short-term wind speed analysis for effective forecasting. Long Short-Term Memory (LSTM) is particularly used in different adapted distinctions with high basic criteria. For instance, Alazab et al [26] used multi-directional LSTM model to forecast stability of power in smart grids. However, if the interactive mechanism is not needed, it still needs a lot of resources to converge neural networks quickly as the existing improved version does not match hardware power acceleration [27]. Lu et al [28] proposed an encoder-decoder LSTM model for wind power estimation by mapping wind power time series data into fixed-length representations. The results showed that the LSTM auto-encoder based preprocessing can perform better compared to simple LSTM wind power forecasting.

The Fourier representation of linear and steady-state analysis approaches focus on optimal filtering using Variation Mode Decomposition (VMD) and Single or Multi-Kernel Regularized Pseudo Inverse Neural Network (MKRPINN) [29]. An Ensemble Empirical Mode Decomposition (EEMD) [30] further decreases the impact of projected modeling of pseudo wind power data which is also scalable to data reduction without any need for further specifications. Different parameter fine-tuning has the additional advantage to acquire useful data characteristics [31]. The one-hour forecasting of seven locations of ground radiation showed that the wind power forecasting performance can be improved by 30% compared to traditional benchmark estimates. Fu et al [32] presented a multi-step ahead technique based on RNN combined with LSTM or GRU also known as Gated Recurrent Unit to improve wind power forecasting accuracy. Shao et al [33] further combined Infinite Feature Selection (Inf-FS) with RNN to overcome short-term wind power forecasting problems. Analysis of data from the US National Renewable Energy Laboratory (NREL) reveals that the efficiency of short-term wind energy forecasts in spring, summer, autumn, and winter has been greatly improved by 11 percent, 29 percent, 33 percent, and 19 percent respectively. However, the RNN has one weakness associated with the high power of the matrix caused by its vanishing gradient. As a result, it is difficult to determine the long-term dependence of the time series in wind power forecasting. However, it has been found that the average error rate and the maximum error rate of LSTM-RNN are lesser than other methods [34]. Lai et al [35] proposed Long and Short Term Times Series Network (LST-Net) to resolve the accuracy problem of time-series forecasting. The empirical evaluation showed that the earlier evaluation of repeated trends in time series data can improve the overall forecasting accuracy.

A conceptual hybrid method associated with Wavelet Packet Decomposition (WPD) is proposed by Liu et al [36], which is a combination of CNN and LSTM to minimize the effect of non-stationary raw data for short-term wind power forecasting. WPD decomposes initial wind speed data based on time series into multiple sub-layer levels where CNN and CNN-LSTM were used for high-frequency and low-frequency evaluation. The empirical evaluation of various test samples has shown that hybrid modeling of various wind speeds can improve the precision of short-term wind power forecasting. Chen et al [37] presented another hybrid approach based on LSTM and an evolutionary algorithm named ELM for wind speed and power forecasting. The empirical evaluation of the four evaluation parameters found that the preferred hybrid approach had achieved the predicted outcomes from the estimation criteria, while the projected results had an actual benefit over the forecasting accuracy. Adopting several models as the mainstream model can only improve the aggregate average forecasting performance. Khodayar et al [25] proposed a graph-based deep neural network to analyze unguided high wind positions consisting of LSTM and graph based CNN model to overcome short-term wind speed forecasting errors. CNN-based deep learning models are adaptable and have been widely used in forecasting studies. For instance, Vasan et al [38] used an ensemble CNN model to improve forecasting accuracies. The deep learning architecture (GCDLA) captures the temporal characteristics of the wind forecasting process. A study of GCDLA findings has shown that hybrid approaches paired with separate neural networks can improve the predictive accuracy of wind speeds. Chang et al [39] developed an improved deep learning network by selecting input features with suitable interfaces and established two types of probabilistic forecasting models. In previous studies, it was reported that the absence of appropriate input features and lack of analysis and selection causes a negative impact when forecasting is applied to high-dimensional wind power data from multi-input. This requires a huge number of computational resources that can also influence usability. Wang et al [40] suggested an ensemble approach to resolving such probabilistic wind power forecasting issues. Time-frequency classification including distinguishing feature extraction for various wavelet transformations along with CNN is processed to achieve desirable estimation. The empirical evaluation showed that some uncertainties in wind power data can be identified using ensemble approaches. Furthermore, Xiao et al [41] proposed hybrid modified architecture centered upon Bat Algorithm (BAT) in combination with Conjugate Gradient (CG) process in order to forecast wind speeds.

In recent studies, Lin and Liu [42] used high frequency SCADA data to forecast wind power of Levenmouth offshore wind turbine. The outliers were detected and removed from data using isolation forest filtering while a deep learning model is finetuned to forecast offshore wind power. Although the isolation forest filtering needs limited memory, it does not employ any distance or density measures to eliminate outliers. As a result, the deep learning model may be affected when applied to multiple offshore wind turbines. Zameer et al [43] used an ensemble ANN model along with genetic programming to overcome instability problem of wind power. The proposed approach was tested on five windfarms and produced reasonable outcomes. Devi et al [44] also used ensemble strategy mainly focused on improving the forecasting performance using LSTM-EFG model combined with cuckoo search optimization and ensemble empirical mode decomposition. In general, the ensemble methods are quite helpful in improving forecasting results by combining multiple models but their improved performance is due to the reduction in the variance component of forecasting errors generated by the participating models. Yildiz et al [45] used variational mode decomposition (VMD) to convert wind power features into RGB images. The image data was then used as input for CNN model to perform short term power forecasting. The accuracy of image-based forecasting is effective but image pixels are not true geographical objects due to limited pixel topology. Therefore, spectral and spatial effects of images on CNN may further needed to be elaborated for effective wind power forecasting. Acikgoz et al [46] used extreme machine learning (EML) strategy to forecast terrain-based wind power on one year data in turkey. The empirical evaluation of seasonal performance showed improvement compared to tradition ANN with minimal forecasting errors. However, the training and testing were conducted on k-folds, which means that the training algorithms had to be ran many times, which may be computationally expensive for large datasets. Niu et al [47] used sequence-to-sequence modeling and attention-based GRU network to improve accuracy and stability of traditional wind power forecasting. GRU is an alternative and improved version of LSTM but computational time of deep learning process increases as attention mechanism applied to wind power forecasting. The summarized comparison of recent studies on wind power forecasting is shown in Table 1.

**Table 1:** Neural networks and input features used in previous recent studies.

Reference, (Year)	Training Strategy	Input Features	Performance Indicator
Zameer et al [43], **(2017)**	ANN and Genetic Programming	Wind Speed, Wind Direction etc.	RMSE: 0.117575
Liu et al [48], **(2018)**	Elman neural network & LSTM	Wind Speed, Wavelet Transformation Features	MAE: 0.44% - 0.90% RSME: 0.62% - 1.11%
Lin and Liu [42], **(2020)**	Isolation Forest Filtering & DNN	Wind Speed, Wind Direction, Pitch Blade, Air Density etc.	MAE: 374.41 RMSE: 517.33
Devi et al [44], **(2020)**	Cuckoo Search Optimization, LSTM-EFG, EEMD	Wind Speed, Wind Power datapoints,	MAE: 4.326 RMSE: 4.387
Acikgoz et al [46], **(2020)**	Extreme Machine Learning, ANN, K-folds	Wind Speed, Wind Direction, Electricity Frequency, Rated Power	NRMSE: 7.01% - 13.09%
Niu et al [47], **(2020)**	Sequence-to-Sequence Modeling, Attention-based GRU	Wind Speed, Wind Direction, Air Density, Air Pressure etc.	NRMSE: 11.28%
Yildiz et al [45], **(2021)**	Variational Mode Decomposition, CNN	Wind Speed, Wind Power, Rated Power, RGB Converted Images	MAE: 0.0376–0.0493 RSME: 0.0499–0.0631

As discussed in the literature study, classifier training is necessary from the beginning for offshore wind power forecasting, which is also a time-consuming practice. Secondly, ensemble-based deep learning strategies for wind power forecasting utilize two or more DNN models, with one predictor propagating forecasting outcomes while others improving the forecasting error. Additionally, the wind speed is more diverse at offshore regions compared to land-based installations, thus observations may contain a significant quantity of outlier values. As a result, eliminating all outliers may have a negative effect on the reliability of wind power forecasting strategies. Instead, our proposed model employs deep auto-encoders to efficiently learn low-dimensional data ensuring minimal reconstruction error. Furthermore, training several layers with a deep autoencoder is more efficient than training single huge transformation based on dimension reduction schemes. As a result, the neural network’s time-consumption problem can be handled more precisely. In particular, a deep auto-encoding based hybrid CNN-LSTM model can facilitate rapid feature analysis and estimation of wind power forecasting.

## Overall methodology for wind power forecasting

**Fig 1:**
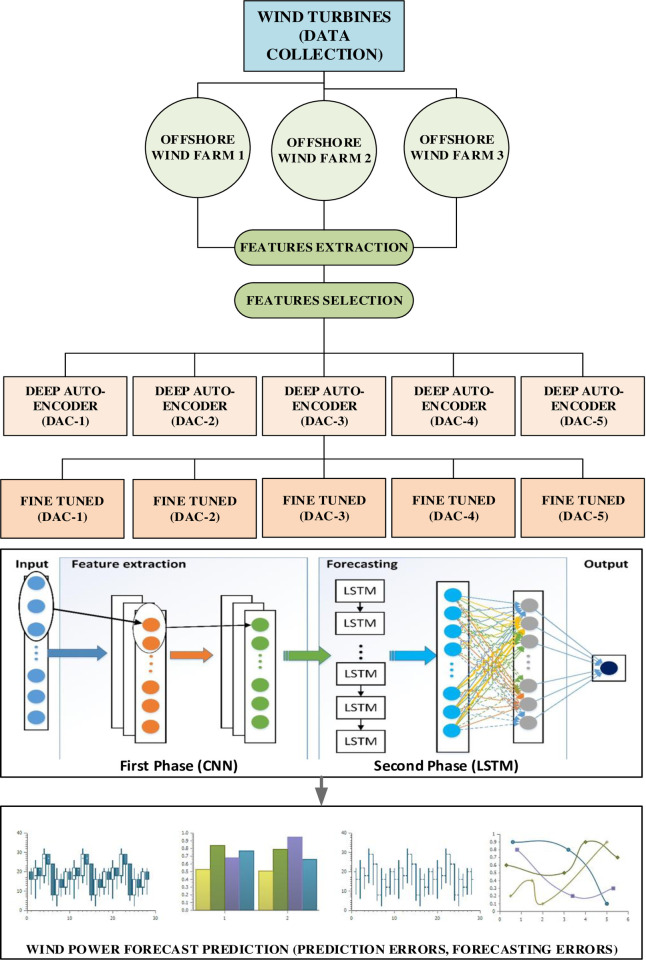
Overall methodology and overview of offshore wind power forecasting framework.

Due to the turbulent and diverse real-time behavior of wind signals, predicting accurate wind power is a difficult task. In order to evaluate the predictability of wind power, the raw wind data is obtained from three separate offshore Windfarms. Next, the raw wind data is analyzed and the valuable variables are chosen that can hold worthy information from the vast range of offshore wind turbines. In all three data sets, a sequence of five Deep Auto-Encoders (DAC) is used to exploit hidden information and focus on meaningful features that can facilitate the predictability of deep learning models. Our proposed deep learning wind power forecasting methodology can efficiently compress and encode wind data, then realizes data in minimized encoded form towards a classification model. Furthermore, auto-encoders has been applied for dimensional reduction that can effectively improve forecasting accuracy. The trained model is then transferred to the deep learning model based on Convolutional Neural Network (CNN) combined with LSTM to predict wind power on all three offshore Windfarms. Finally, the efficiency and effectiveness of the proposed model over different offshore Windfarms are evaluated in terms of prediction accuracy and forecasting errors as shown in Fig 1.

### Deep Auto Encoder (DAC)

DAC is a simple deep auto-encoder and feedforward artificial neural network consisting of three basic layers, i.e., the entry layer also known as the input layer the hidden layer responsible for applying weights using the activation function, and the output layer to produce final results. Each node in the neural network except input nodes is a neuron that utilizes a non-linear activation function. Several levels, including non-linear stimulus, discriminate against the Hidden layer by a standard DAC classifier to differentiate non-linearly separable data [23,49].

DAC auto-encoders each with one hidden layer and hidden weights minimize the squared error with a quadratic penalty (weight decay). Contractive auto-encoder (CAE) is also an alternative to the weight decay DAC strategy. We used a specific normalization that has the advantage of robustness with slight deviations around the test points to favor interfaces that converge more precisely on the training set. Like the Contractive auto-encoder, the DAC auto-encoder can obtain a comparable measure of regularization on objective function distribution as shown in Eq 1.


$$J_{CAE}=\sum_{x\in D_{n}}\left(L(x,g(f(x)))+\lambda ∥J_{f}(x\right){∥}_{F}^{2})$$ (1)

Where the approximation error is L, λ hyper-parameter controls the regularization power, $J_{f}(x){∥}_{F}^{2}$ promotes the estimation with training data across suburb as a constructive for feature space.

#### Correlation between DAC and weight decay

Since it becomes obvious to assume Frobenius standard of Jacobian squared corresponds towards an L2 weight decay (for instance, an activation function *s*_*f*_). Having lower weights through a static scenario is also the best solution towards reduction. However, in the case of the sigmoid non-linear activation function, reduction and robustness can also be achieved by placing hidden units into their saturated state. If an encoder represented as *f* function transforms the source $x\in R^{d_{x}}$ to a hidden $h(x)\in R^{d_{h}}$ representation, then the resulting form can be expressed as Eq 2.


$$h=f(x)=s_{f}(W_{x}+b_{h})$$ (2)

Here *s*_*f*_ is a type of sigmoid non-linear activation function, while the *d*_*h*_×*d*_*x*_ weight matrix W, and a bias vector $b_{h}\in R^{d_{h}}$ are parametrized towards the encoder.

The key features of the proposed auto-encoding strategy are as follows:

1. It renders the encoding less vulnerable from its training dataset to slight alterations.
2. Encoding is achieved by utilizing a regularization or penalty scheme to an objective function.
3. The overall outcome is to minimize the sensitivity of the learning representation against the training feedback.
4. Encoder activation sequences are regularized and must comply with the Frobenius norm of the Jacobian matrix concerning input data.
5. The DAC auto-encoder is generally used similar to other auto-encoders by activating only when the data point is not labeled by other encoding schemes.

**Fig 2:**
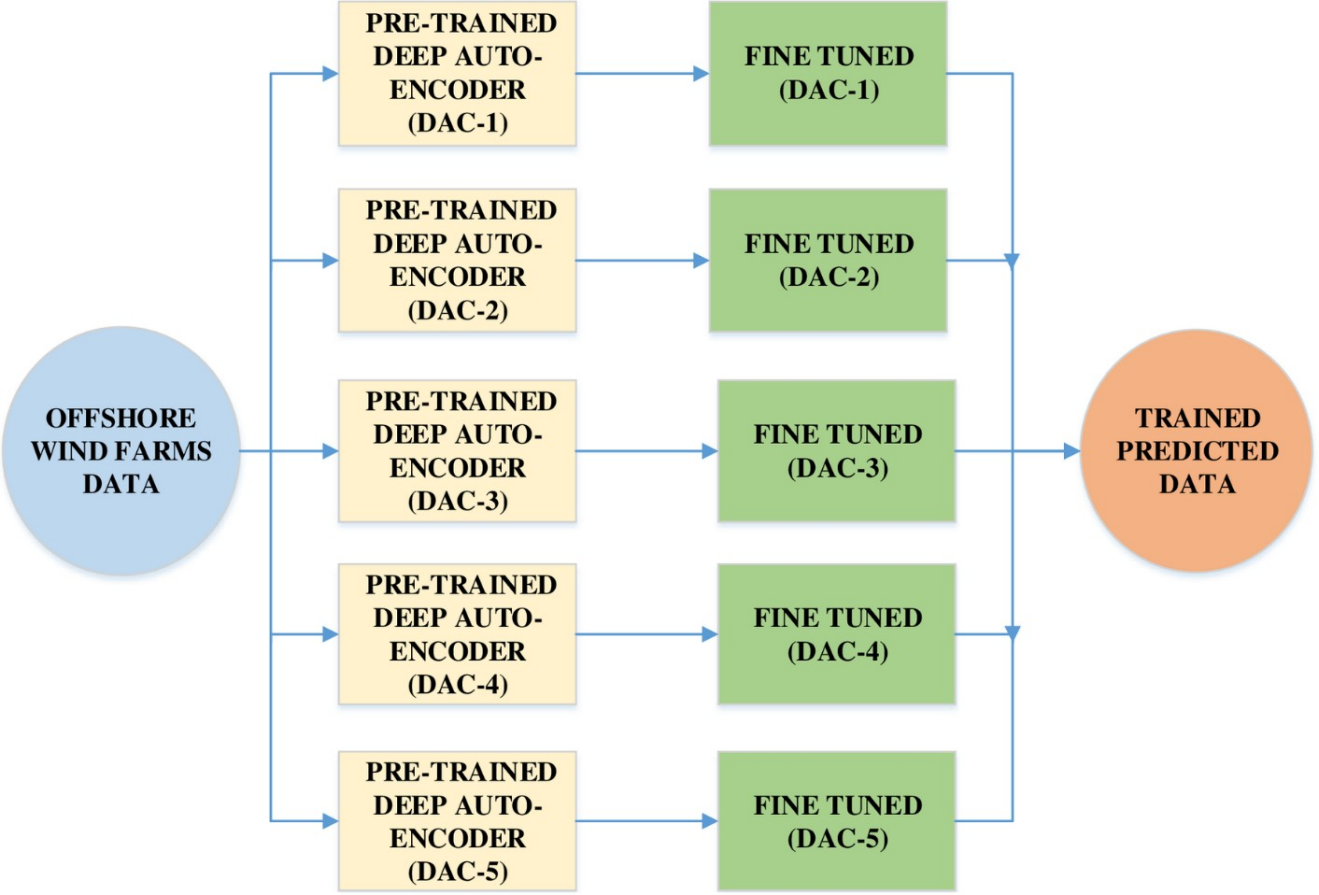
Dataflow graph of deep auto-encoders used in pre-processing of wind turbine datasets before actual wind power forecasting.

The unpredictable and unstable nature of the wind makes it very difficult to extract certain wind patterns in order to precisely forecast reliable wind energy. The instability of wind contributes to the wide range of training samples that have a significant effect on the precision of power prediction. First, a group of five DAC deep auto-encoders is used in all three Offshore Windfarm datasets to extract hidden features and meaningful data patterns in a low-dimensional space. DAC auto-encoder is an unsupervised neural network that learns how to compress and encode information efficiently, and then learns how to reconstruct the features of a reduced encoded representation roughly similar to the actual input. The dataflow diagram of the proposed DAC based Deep Auto-Encoding framework is shown in Fig 2. In the proposed auto-encoder framework, the dimensionality reduction strategy is also used for data to further improve the efficiency of wind power forecasting during pre-processing wind data.

### Offshore Windfarm (NREL) dataset

**Fig 3:**
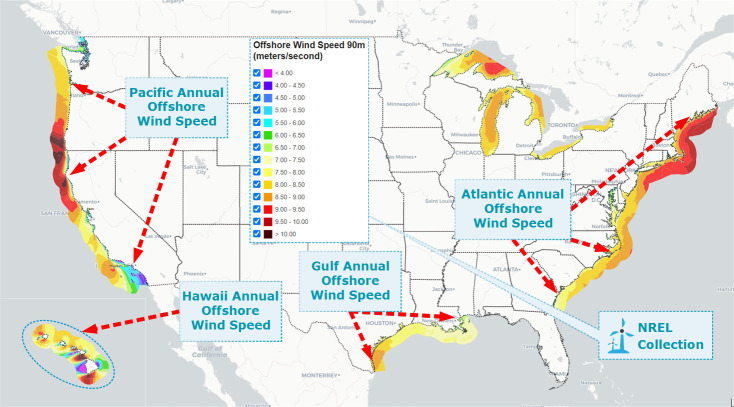
Wind speed is observed on an annual basis in several regions where offshore wind turbines were positioned. The NREL Maps API (https://maps.nrel.gov/) and the Offshore annual wind speed data are utilized to generate a geospatial map of offshore regions.

Windfarms data is pre-processed based on offshore wind turbine data from U.S. NREL repository consisting of multiple offshore regions [50]. Datasets provide wind potential and real-time measurements of wind turbines for certain intervals located in different offshore regions. The first dataset is processed based on a scale of 30 meters to 90 meters with a measurement duration of 1 hour, 7 hours, and 12 hours, including the climatic data of 164,000 Windfarms. The second Windfarm data is based on the Hawaii region of the United States, with an average grid of 2 kilometers recorded during the month of January. Additionally, the third Windfarm data is based on an offshore wind analytics database compiled via different wind speed design parameters. Moreover, the real-time wind data are evaluated for 17 years only by the MERRA time series, and the various meteorological specifications. Fig 3 shows the annual wind speed measured by the NREL on different offshore regions of the United States. Higher wind speeds indicate the potential of power generation by offshore wind turbines in the United States. A significant number of offshore windfarms are located in these regions, that are also utilized in wind power forecasting analysis.

### CNN-LSTM neural network architecture

**Fig 4:**
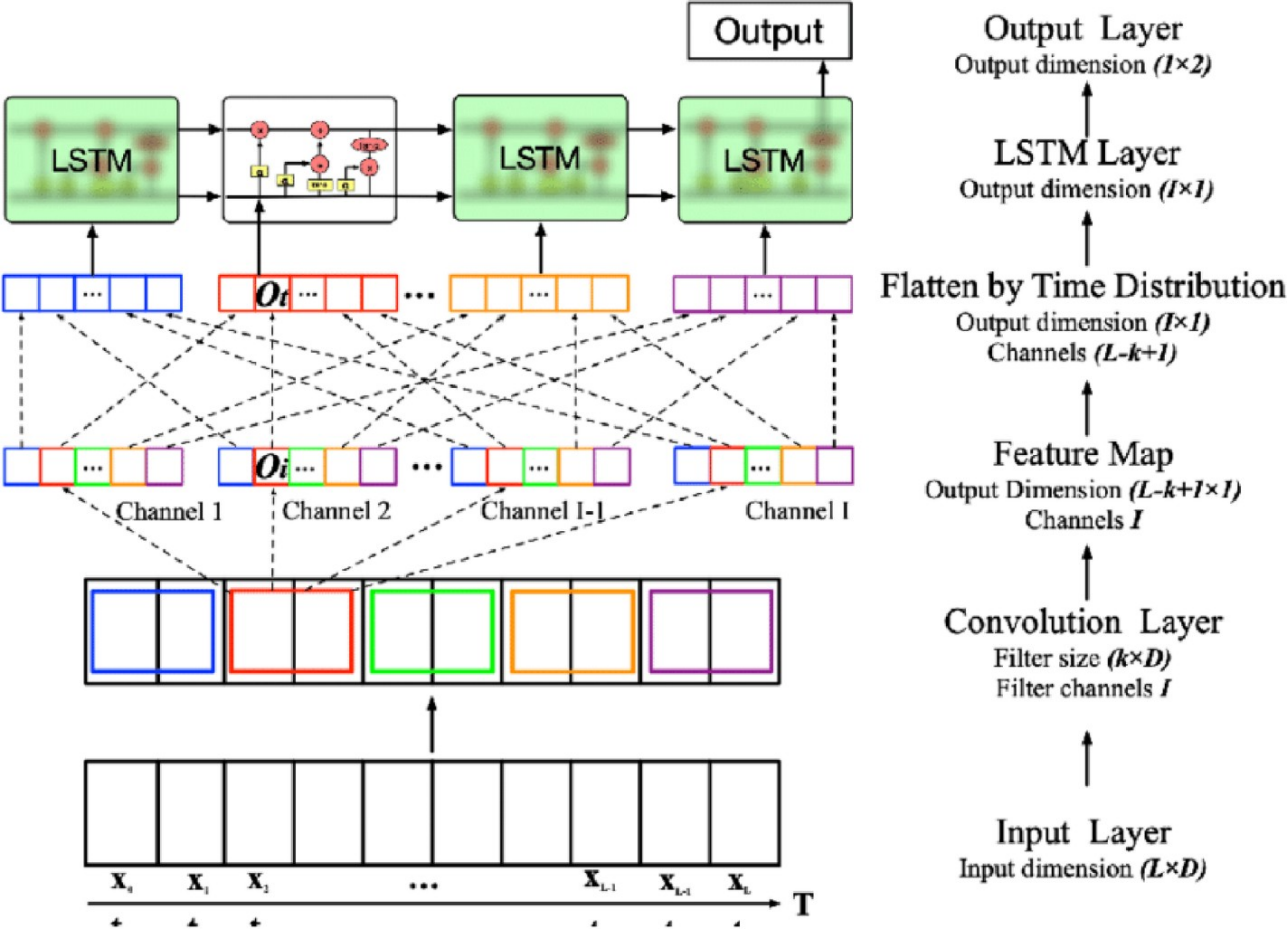
CNN-LSTM neural network architecture.

The pre-trained model is then transferred towards a CNN-LSTM neural network for offshore wind power forecasting. The CNN-LSTM framework is used as the primary indicator throughout this research. The proposed Convolution Neural Network strategy has succeeded in reducing the effect of computational complexity and has also achieved significant improvement in extracting and generalizing features [51]. LSTM is capable of processing 1-decision statistical analysis as well as in making assumptions by generating an outcome for each timespan. In order to isolate the features of precompiled results, a 1-D convolutional layer along with the LSTM framework is implemented in this paper as a major indicator. The designated CNN-LSTM configuration with the addition of CNN layers in the LSTM model is shown in Fig 4. The LSTM layer along with two dense layers and three fully connected layers is configured, while max-pooling is also used within the hybrid CNN-LSTM model. Through multiple tests, the activation function of each convolutional layer is calculated against Parametric ReLU (PReLU), then the sigmoid function is estimated to activate that LSTM layer. Moreover, zero paddings have been used only between different components of convolution layers to preserve a certain proportion.

Indeed, the convolution layer is the main focus of the convolution neural network. Within this layer, there are also two main processes, local independent correlation and temporal classification of outputs [52]. To further simplify the basic calculations, the former calculation is also adopted for the calculation of each filtered relevant information. Although described, the performance of the convolution layer may be enforced [53]:

$$h^{k}=f((W^{k}*x)+b_{k})$$ (3)

Where * signify convolutional process, f (·) is the activation function, *W*^*k*^ and *b*_*k*_ are the weights and biases of the k^th^ function.

### LSTM (long-short term memory) network

LSTM is more resourceful relative to conventional Recurrent Neural Network (RNN). However, based on the input gate, the forget gate, and even the corresponding output, the same weight of perception appears to be malicious [54]. The defined specific tasks can be observed as follows:

1. How data are excluded again from the convolutional layer is decided by the forget gate.
2. It will change the cell state, but how much additional data throughout the input gate will be applied to the main contribution.
3. A sigmoid function is executed as an output gate, and the cell state is analyzed by an activation function that pairs with the sigmoid output layer to produce the desired total output [55].

Although there four key LSTM parameters such as internal memory state *c*_*t*_, forgot gate *f*_*t*_, input gate *i*_*t*_, and output gate *o*_*t*_. The computation phase at each component is represented using Eqs 4–7 at period t time:

$$f_{t}=\sigma (W_{f}.[x_{t},h_{t-1}]+V_{f}.c_{t-1}+b_{f})$$ (4)

$$i_{t}=\sigma (W_{i}[x_{t},h_{t-1}]+V_{i}.c_{t-1}+b_{i})$$ (5)

$$c_{t}=c_{t-1}*f_{t}+i_{t}*\tanh(W_{c}[x_{t},h_{t-1}+b_{c})$$ (6)

$$o_{t}=\sigma (W_{o}.[x_{t}+h_{t-1}]+V_{o}.c_{t}+b_{o})$$ (7)

$$h_{t}=o_{t}*\tanh(c_{t})$$ (8)

For offshore Windfarm datasets, optimum fine-tuned specifications are shown in Tables 1–3, accordingly. In the hidden layers, the number of neurons ranges from 50 to 250, whereas the number of epochs varies from 200 to 400. All offshore Windfarm datasets are pre-trained and saved as train models. The training model is then used in the proposed LSTM-CNN deep learning model to predict wind power for all three offshore Windfarms. For the input nodes, the Rectified Linear Unit (ReLU) is used. In deep learning models, ReLU is the most widely selected activation function. This activation function returns 0 if any negative response is obtained, and retains input for any positive value of x. It is numerically represented using Eq 9.


$$f(x)=x^{+}=\max(0,x)$$ (9)

**Table 2:** Precise fine-tuned parameters for Offshore Windfarm 1.

Layer No.	No. of Neurons	Maximum Epoch	L2 Weight Regularization	Dropout Ratio
1	150	200	0.00002	0.2
2	125	175	0.00001	0.1
3	100	150	0.00001	0.1
4	100	125	0.00001	0.1
5	70	100	0.00001	0.1

**Table 3:** Precise fine-tuned parameters for Offshore Windfarm 2.

Layer No.	No. of Neurons	Maximum Epoch	L2 Weight Regularization	Dropout Ratio
1	150	200	0.00003	0.2
2	125	200	0.00001	0.2
3	100	175	0.00001	0.1
4	100	150	0.00001	0.1
5	90	125	0.00001	0.1
6	70	100	0.00002	0.1
7	60	100	0.00002	0.1

Where the input of the neural network is x also termed as a ramp function identical to split-wave rectification.

The Softmax activation function is used with the output layer to evaluate forecast errors which is also a multi-dimensional generalization of the logistic equation. It can be used for multiple regression analysis and can also be used as the final activation function of the neural network to stabilize the probability distribution performance over the expected output groups [56]. The neural network is generally optimized for output N values for each class of classification model, while the Softmax function can normalize these outputs by transforming the weights to the sum of probabilities. Each value in the output of the Softmax function is interpreted as the probabilities of being a member of each class. It is statistically represented using Eq 10.


$$\sigma {\left(\vec{z}\right)}_{i}=\frac{e^{z_{i}}}{\sum_{j=1}^{K}e^{z_{j}}}$$ (10)

Where *σ* signifies the softmax function, $\vec{z}$ is the input vector, $e^{z_{i}}$ is the standard exponential function for input vector, *K* shows the variety of sections throughout the algorithm for a multi-class, while $e^{z_{j}}$ is a standard exponential function for output vector. In order to build a deep learning model, the entropy function is often used to detect accuracy loss. It considers each tensor as input and targets the same shape of a tensor as an output. The Adam optimizer is used to assemble and configure a deep learning model that is often known as a stochastic descending gradient. Adam Optimizer modifies network weights and determines unique active learning rates for each element of the deep learning architecture [57,58]. The decaying mean (DM) of the pas squared gradient is expressed using Eqs 11 and 12 respectively [59].


$$m_{t}=\beta_{1}m_{t-1}+(1-\beta_{1})g_{t}$$ (11)

$$v_{t}=\beta_{2}v_{t-1}+(1-\beta_{2})g_{t}{}^{2}$$ (12)

Where *m*_*t*_ and *v*_*t*_ are the approximate measures during the first and second moment gradients. As a result, the respective gradient is indicated for each moment. Adam optimizer further counteracts these biases by estimating the bias-corrected first and second moments as shown in Eqs 13 and 14 respectively.


$${\hat{m}}_{t}=\frac{m_{t}}{1-\beta_{1}^{t}}$$ (13)

$${\hat{v}}_{t}=\frac{v_{t}}{1-\beta_{2}^{t}}$$ (14)

Sparse Categorical Cross-entropy is used to compare the predicted label and true label to calculate the accuracy loss and validation errors. Sparse Categorical Cross-entropy is an arithmetic variant of the categorical cross-entropy loss function which does not require the transformation of target labels into category formats [60]. Mathematically, the relationship between different parameters of the loss function can be expressed using Eq 15.


$$J(w)=-\frac{1}{N}\sum_{i=1}^{N}[y_{i}\log({\hat{y}}_{i})+(1-y_{i})\log(1-{\hat{y}}_{i})]$$ (15)

Whereas *w* indicates the parameters of the deep learning model such as weights of the neural network, while *y*_*i*_ represents the true label and ${\hat{y}}_{i}$ becomes the predicted label respectively.

Following the formation of the CNN-LSTM model, the number of hidden layers, neurons, and operational parameters are fine-tuned for each offshore Windfarm. Tables 2–4 shows the precise fine-tuned settings used in power forecasting of Windfarms.

**Table 4:** Precise fine-tuned parameters for Offshore Windfarm 3.

Layer No.	No. of Neurons	Maximum Epoch	L2 Weight Regularization	Dropout Ratio
1	150	200	0.00003	0.2
2	140	175	0.00001	0.2
3	120	150	0.00001	0.1
4	100	150	0.00001	0.1
5	100	125	0.00001	0.1
6	90	125	0.00002	0.1
7	60	100	0.00002	0.1
8	50	100	0.00002	0.1

## Results and discussion

The Keras Interface integrated with the TensorFlow platform makes it easy to transform layers, activation and loss functions, etc. into a variety of prototype deep learning models [61]. It also offers a compilation strategy for customizing the training process in each layer, including losses, optimization, and other built-in learning configurations to train the constructed model [62]. The training is effectively conducted corresponding to the input design, but also dense layers. The training process is automatically performed by TensorFlow corresponding to the input shape of data and dense layers. Deep learning further drives the integration of user-defined enhanced functionality to train feature representation in a short period to solve complex issues.

**Fig 5:**
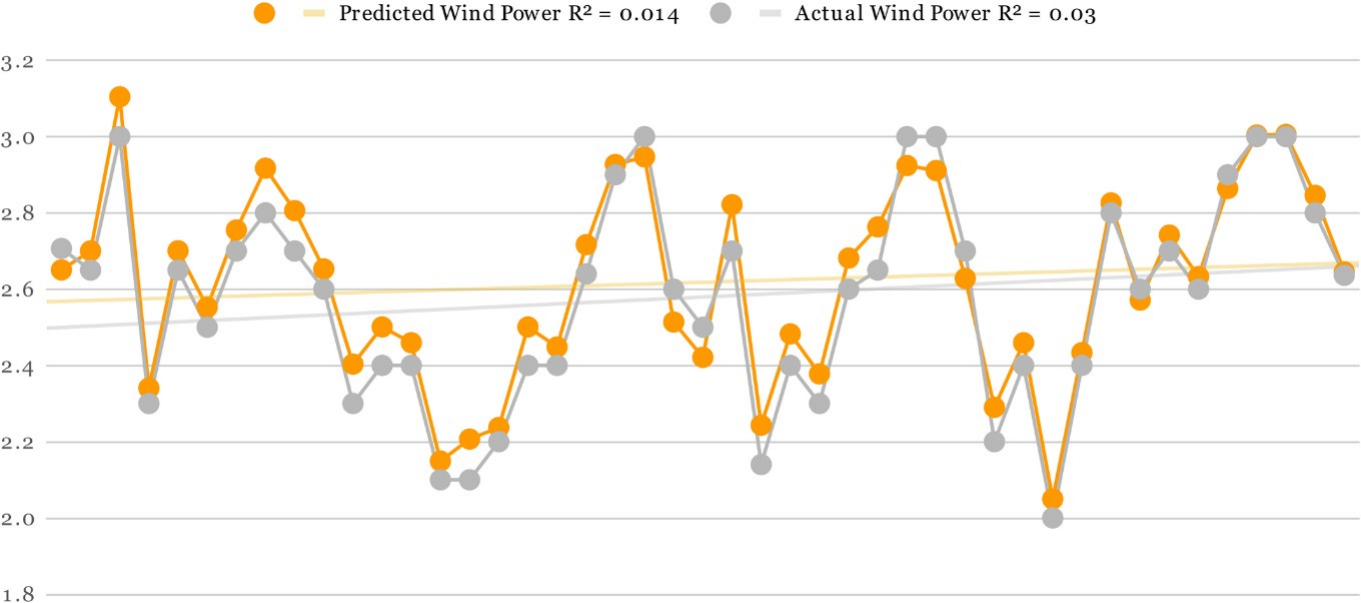
Predicted and actual power generation on Offshore Windfarm 1 based on Table 2 fine-tuned setting and proposed forecasting architecture.

**Fig 6:**
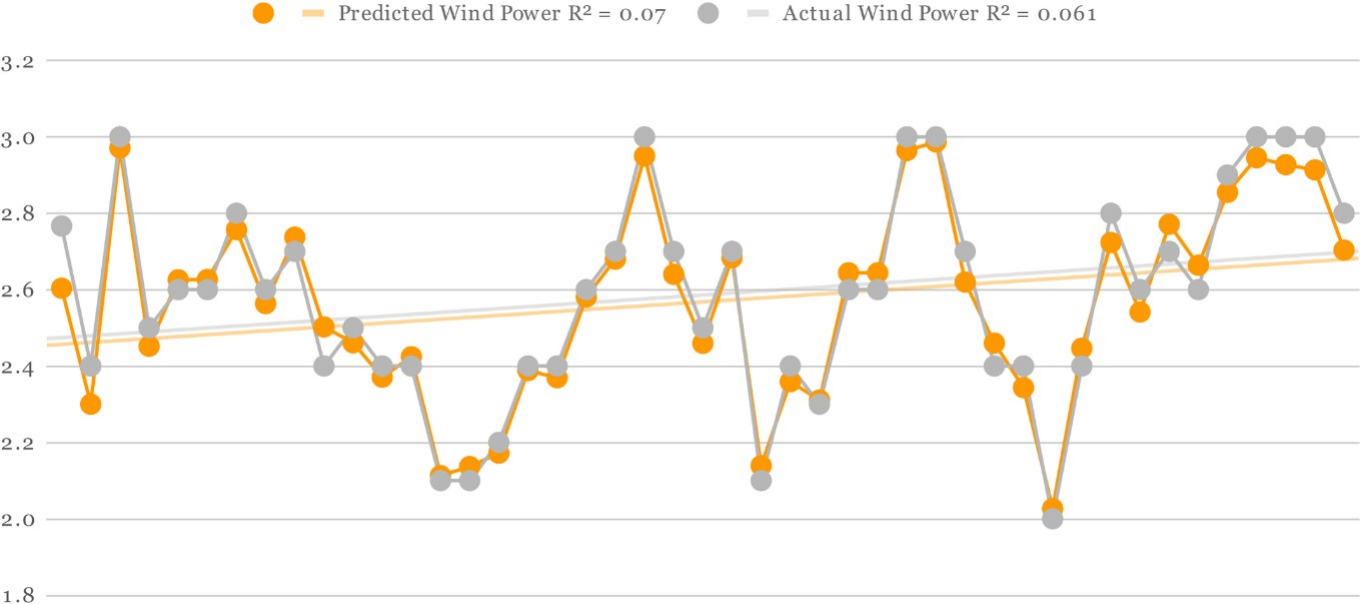
Predicted and actual power generation on Offshore Windfarm 2 based on Table 3 fine-tuned setting and proposed forecasting architecture.

**Fig 7:**
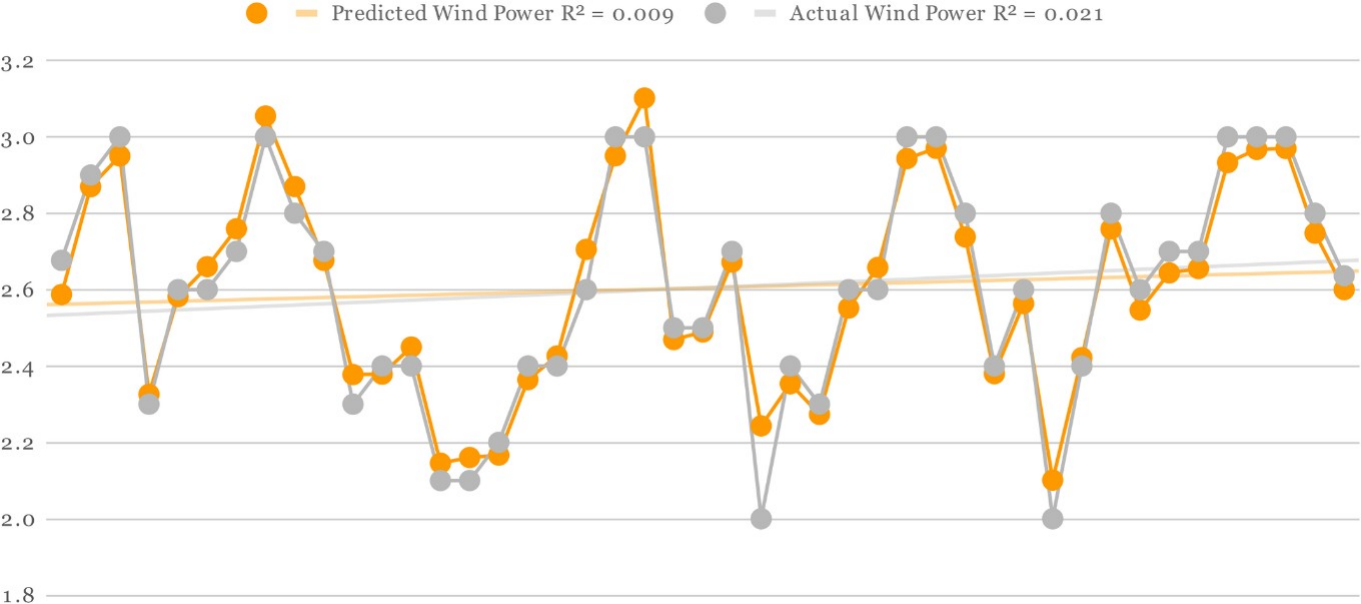
Predicted and actual power generation on Offshore Windfarm 3 based on Table 4 fine-tuned setting and proposed forecasting architecture.

For all three Offshore Windfarms, the actual and predicted wind power is displayed in Figs 5–7 respectively. The orange curve indicates the predicted power by DAC-CNN-LSTM strategy while the grey curve shows the actual wind power generated from US offshore turbines collected by NREL. In Fig 5, the offshore wind farms predicted values were generated using Table 1 fine-tuned setting. There is a small error margin between actual and predicted wind power which clearly shows effectiveness of proposed methods for offshore wind power forecasting. Furthermore, the R-squared correlation values of predicted and actual wind power are 0.01 and 0.03 also shows a close association between predicted and actual results. In the case of Figs 6 and 7, the predicted and actual R-squared correlation of Offshore Windfarm 2 is 0.07 and 0.06, while the predicted and actual R-square correlation of Offshore Windfarm 3 is 0.01 and 0.02 respectively. The observations in all figures reveal that predicted power is quite comparable to actual power, with minor variations at some intervals. However, the overall consistency between the predicted and actual power shows the flexibility and reliability of proposed model in forecasting wind energy across multiple offshore wind turbines.

**Fig 8:**
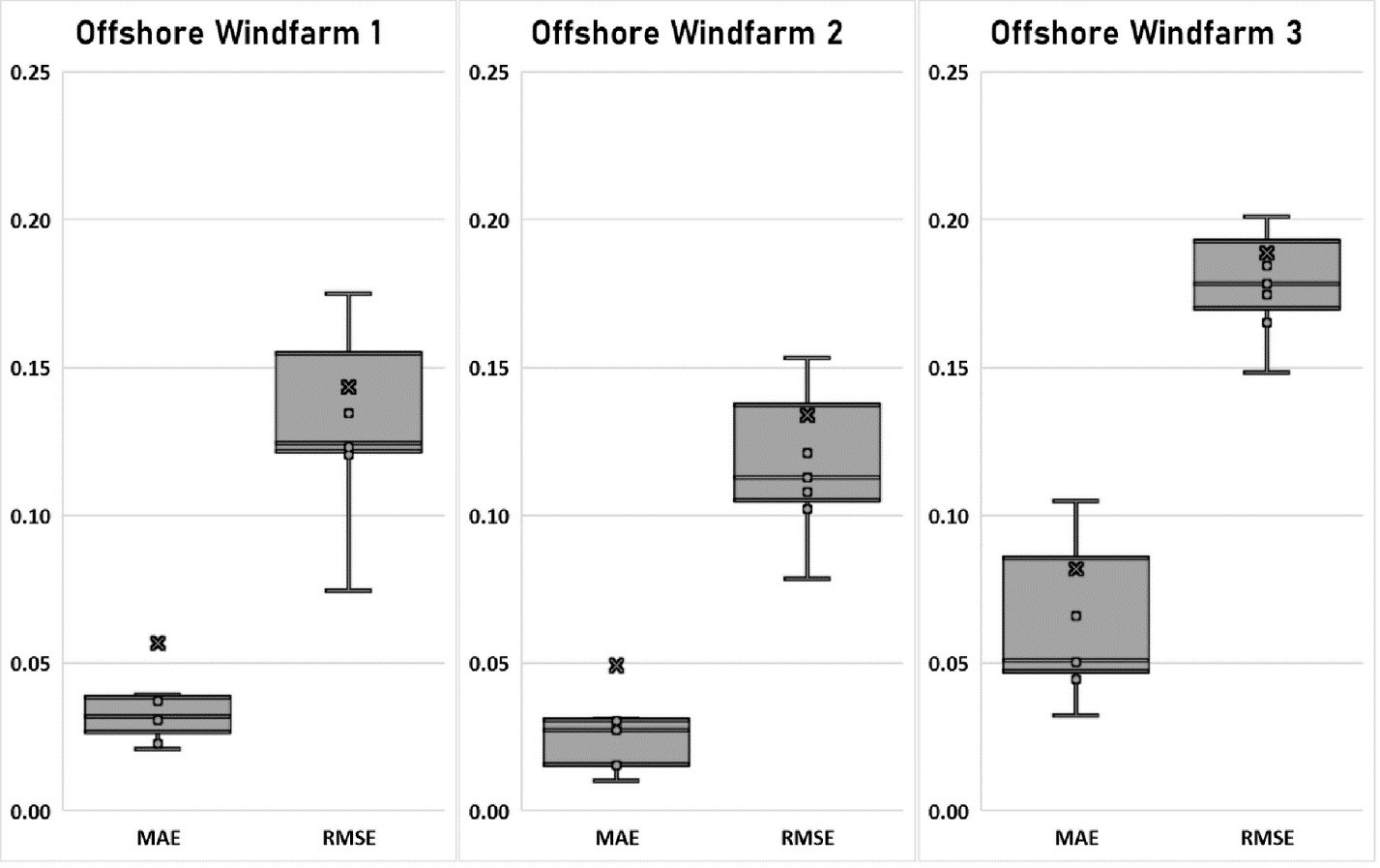
Distribution of forecasting errors (Percentiles) for Offshore Windfarms 1, 2 and 3.

Offshore Windfarms forecasting performance was further assessed in terms of MAE and RMSE errors. The MAE and RMSE errors are widely used to evaluate performance of time-series and other non-label data. In Fig 8, we used boxplots to show the distribution of errors in the form of minimum, maximum and Q1-Q3 percentile ratios based on outcomes generated by deep learning models. The minimum MAE error for all three Offshore Windfarms is between 0.01 to 0.04, while the minimum RMSE for all three Offshore Windfarms is between 0.07 to 0.15 respectively. The RMSE error of DAE-CNN-LSTM is little higher than its MAE error but the overall effectiveness of proposed model is the same. In the case of Q1-Q3 percentile ratios, all Offshore Windfarms MAE and RMSE error is less than 0.20. The lower error percentile ratios show higher confidence level in the evaluation and outcomes of the proposed DAE-CNN-LSTM deep learning model. The low MAE and RMSE error also indicate that the proposed model is quite effective in forecasting offshore wind power regardless of offshore regions.

The proposed model is also fine-tuned to attain the appropriate parameters for individual mean absolute error (MAE), along with root mean square error (RMSE). For fine-tuning the deep learning model, optimization function, the training error rate, activation, and loss specifications were used for training and testing [63]. The (MAE, RMSE) values of Offshore Windfarm 1 are (0.02, 0.0747) and (0.0103, 0.0786) for Offshore Windfarm 2 and (0.0324, 0.1485) for Offshore Windfarm 3 respectively. Numerically, Eqs 16 and 17 were used to measure MAE and RMSE for three Windfarms [64,65].


$$MAE=\frac{\sum_{i=1}^{n}\left|y_{i}-x_{i}\right|}{n}$$ (16)

Where the variable *y* is predicted, *x* is the actual value, *n* is the number of observed data points. Aggregate variations among different components and samples are rooted in terms of RMSE. The predicted value is the first, while the actual value is the second value.


$$RMSE=\sqrt{\frac{\sum_{t=1}^{T}{\left(x_{1,t}-x_{2,t}\right)}^{2}}{T}}$$ (17)

Where expected values are *x*_1,*t*_, *x*_2,*t*_ in observation and *T* is the cumulative number of measurements or observations. The predicted wind power error values further compared with the common state-of-the-art algorithms, i.e., REP Tree, SVM, Random Forest, J48, Back and Forward Procedure Ensemble Selection, and BPNN respectively. Table 5 demonstrates the MAE and RMSE errors for all three Offshore Windfarm datasets for different state-of-the-art wind power forecasting models. In offshore windfarm 1, the MAE and RMSE are improved relative to existing state-of-the-art methods. Comparably, the MAE and RMSE for offshore windfarm 2 achieved better results compared to other windfarms. Additionally, for the offshore windfarm 3, the MAE and RMSE gave better results for the proposed model in comparison with other classification models. The Rep tree and BPNN have improved MAE and RMSE scores (0.0229, 0.1204) for the offshore windfarm 1 dataset, concerning our proposed model. Compared to other approaches, the random forest performs the best MAE and RMSE prediction scores (0.0154, 0.1022) for the offshore windfarm 2 dataset. Other than the proposed DAE-CNN-LSTM model, the classification models such as J48 and ensemble selection showed a strong MAE and RMSE scores (0.0446, 0.1653) for the offshore windfarm 3 dataset. Fortunately, the outcomes of Table 5 showed that our proposed model is more flexible and reliable compared to other state-of-the-art wind power forecasting approaches.

**Table 5:** Comparison of the mean absolute error (MAE) and root mean square error (RMSE) of the proposed model with other state-of-art forecasting methods for all three offshore windfarms.

Dataset Regional Distribution	Errors	Random Forest	J48	REP Tree	SVM	Ensemble Selection	BPNN	Proposed Model
Offshore Windfarm 1	MAE	0.0371	0.0395	0.0229	0.2145	0.0319	0.0307	0.02
	RMSE	0.1231	0.1751	0.1345	0.2520	0.1245	0.1204	0.0747
Offshore Windfarm 2	MAE	0.0154	0.0273	0.0164	0.2135	0.0312	0.0305	0.0103
	RMSE	0.1022	0.1532	0.1129	0.2621	0.1212	0.108	0.0786
Offshore Windfarm 3	MAE	0.105	0.0446	0.0503	0.2242	0.051	0.0661	0.0324
	RMSE	0.1748	0.2011	0.1784	0.2689	0.1653	0.1844	0.1485

Furthermore, the state-of-the-art approaches such as Random Forest, J48, REP Tree, SVM, Back and Forward Method Ensemble Selection, BPNN are also compared to proposed DAE-CNN-LSTM strategy in terms of Normalized Mean Absolute Error (NMAE) and the Normalized Root Mean Square Error (NRMSE) respectively. NMAE and NRMSE are also used to evaluate the effectiveness of times-series or non-label data under normalized scales. Thus, lowering the NRMSE value maximizes the reliability of wind power forecasting models. Table 6 shows the comparison of the proposed model to other state-of-the-art forecasting approaches on-premise of NMAE and NRMSE. It is certain that across all three Offshore Windfarms, our proposed DAE-CNN-LSTM model outperforms existing state-of-the-art methods in terms of NMAE and NRMSE. Offshore Windfarms 1–3 showed (0.0020, 0.0116), (0.0011,0.0102) and (0.0032, 0.0105) normalized errors respectively. The NMAE and NRMSE analyses further demonstrate that the feature engineering and forecasting strategies are precise and quite useful in accurate forecasting regardless of diverse behavior of offshore winds.

**Table 6:** Comparison of the normalized mean absolute error (MAE) and normalized root mean square error (RMSE) of the proposed model with other state-of-art forecasting methods for all three offshore windfarms.

Dataset Regional Distribution	Errors	Random Forest	J48	REP Tree	SVM	Ensemble Selection	BPNN	Proposed Model
Offshore Windfarm 1	NMAE	0.0051	0.0045	0.0033	0.0315	0.0052	0.0049	0.0020
	NRMSE	0.0216	0.0305	0.0201	0.0425	0.0252	0.0167	0.0116
Offshore Windfarm 2	NMAE	0.0014	0.0029	0.0026	0.0309	0.0038	0.0027	0.0011
	NRMSE	0.0143	0.0228	0.0161	0.0366	0.0174	0.0139	0.0102
Offshore Windfarm 3	NMAE	0.0105	0.0057	0.0061	0.0308	0.0319	0.0108	0.0032
	NRMSE	0.0222	0.0247	0.0260	0.0356	0.0213	0.0238	0.0105

**Fig 9:**
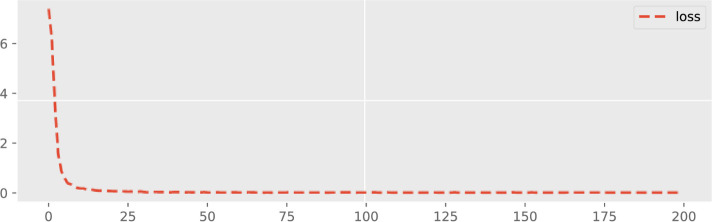
Validation loss across 200 epochs for Offshore Windfarm 1 based on adopted fine-tuned forecasting settings.

**Fig 10:**
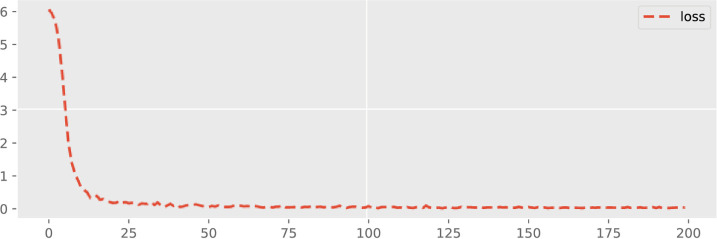
Validation loss across 200 epochs for Offshore Windfarm 2 based on adopted fine-tuned forecasting settings.

**Fig 11:**
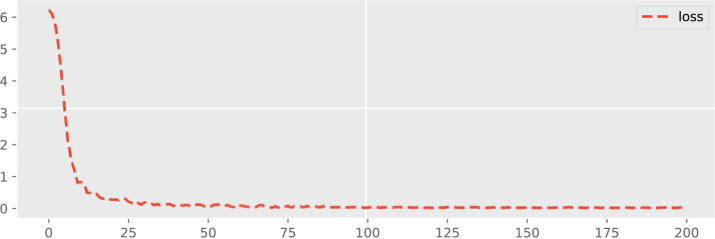
Validation loss across 200 epochs for Offshore Windfarm 3 based on adopted fine-tuned forecasting settings.

The dynamic forecasting plot of all three Offshore Windfarms (1, 2 and 3) in terms of loss configuration is given in Figs 9–11. The epoch values are provided on the x-axis and also the accuracy loss amongst all three Offshore Windfarm datasets is specified on the y-axis. In offshore windfarm 1, the loss curve starts at a high range on the y-axis, and loss becomes lowest and stable approximately after the 25 epochs. Similarly, the loss curve for offshore windfarm 2 starts at a high range on the y-axis, and approximately after the 50 epochs, it becomes stable and behaves in the same direction. Lastly, for the offshore windfarm 3, the loss curve starts from a high range on the y-axis and after 50 epochs, the curve is in a linear direction with the lowest and stable loss. The accuracy loss figures are generated by fitting the trained data against test data. The linear curve with fewer error losses indicates the effectiveness of the proposed DAE-CNN-LSTM model on the data used for both training and testing sets. In general, the validation loss shows how much a model is affected by error generated by forecasting models. Low validation error indicates the higher stability of forecasting model for different epoch intervals.

**Fig 12:**
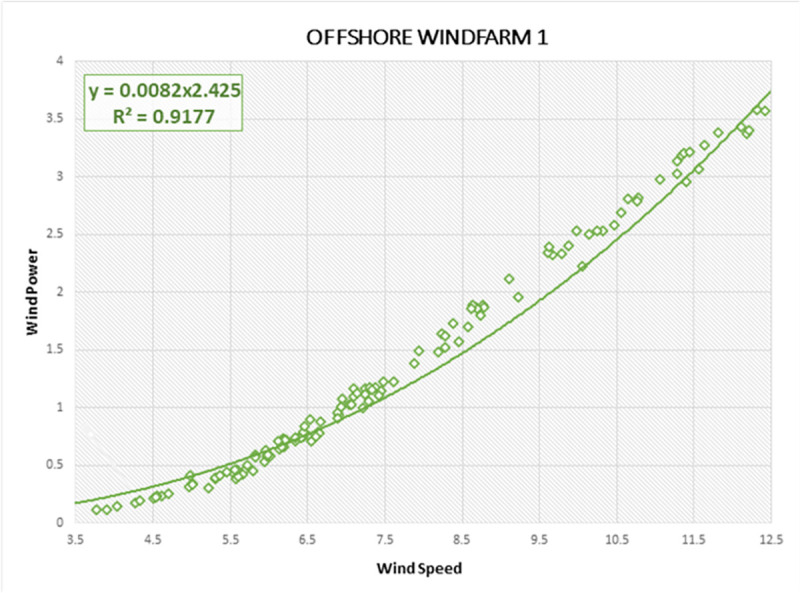
R-Squared correlation and best curve fitting on wind power and wind speed observations of Offshore Windfarm 1 dataset.

**Fig 13:**
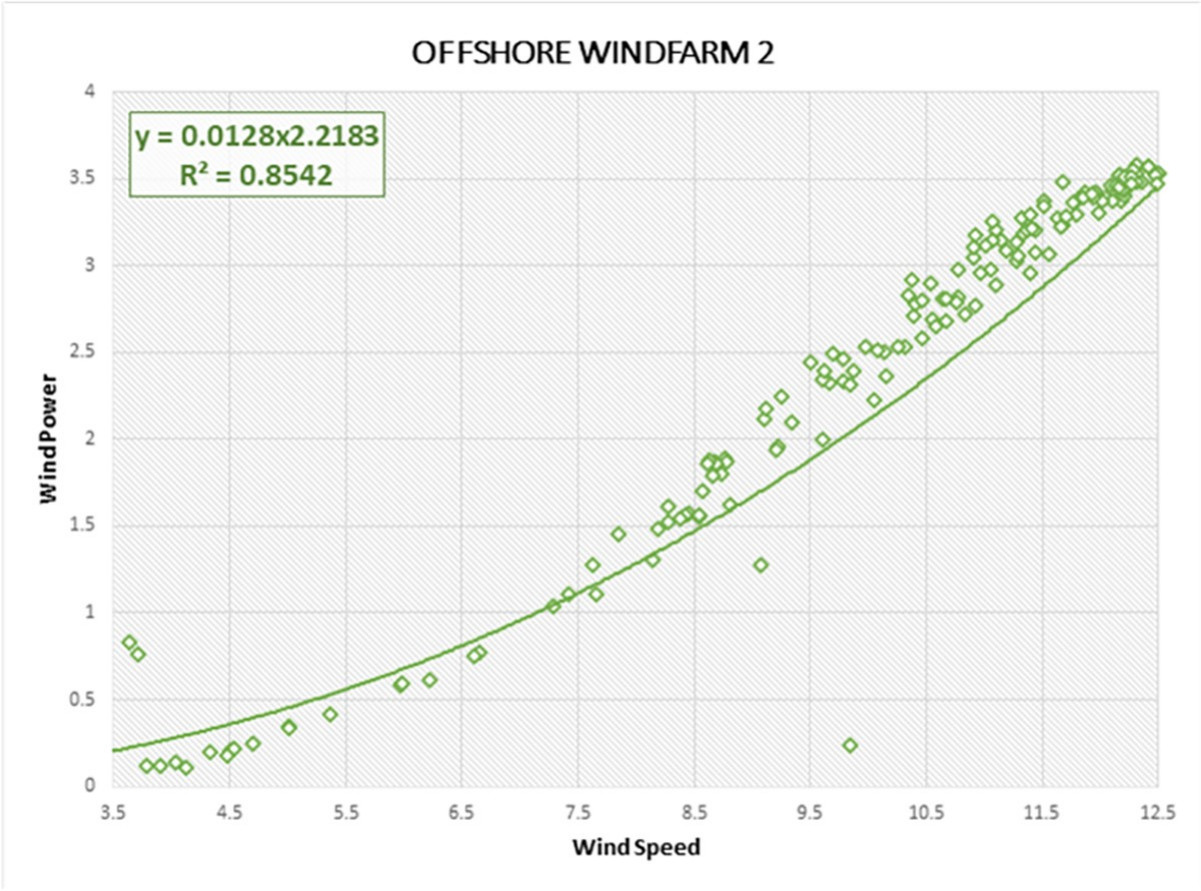
R-Squared correlation and best curve fitting on wind power and wind speed observations of Offshore Windfarm 2 dataset.

**Fig 14:**
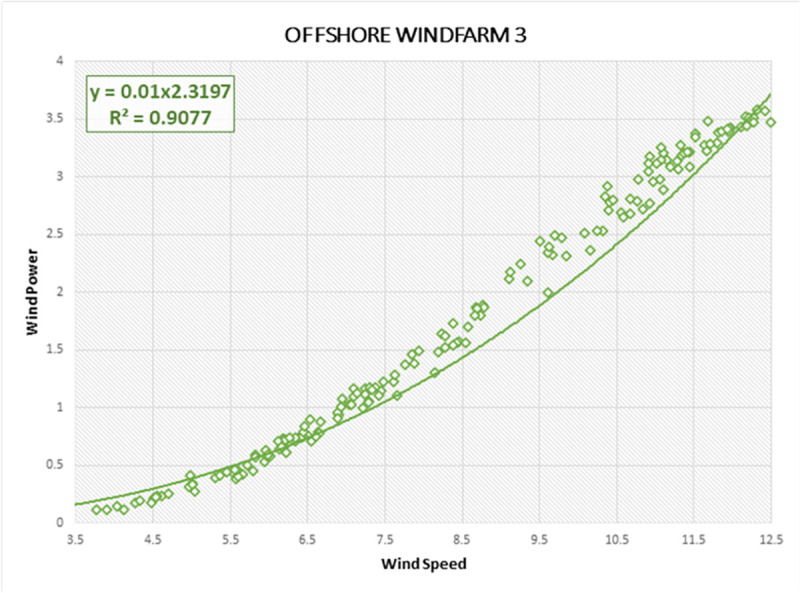
R-Squared correlation and best curve fitting on wind power and wind speed observations of Offshore Windfarm 3 dataset.

Lastly, the R-Squared correlation is used to illustrate the association of wind data characteristics and actual wind power generated by offshore regions. High association between predictor (wind power) and response variable (wind speed) indicates the probability of accurate forecasting on Offshore Windfarms by forecasting models. R-squared describes the measure by which the uncertainty with one component influences the fluctuation for the other component. It is a statistical measure of how the regression line is correlated with wind power forecasting results [20]. The R-Squared curves are estimated for all three Offshore Windfarms, as shown in Figs 12–14, respectively. The vertical line displays the predicted values in the model within each curve, whereas the horizontal line indicates the attributes of the wind data as observed values. The green dots comprise the specification and the dynamics of the wind data as well as the linear regression curve depicts a model strength dependent on R-Squared. We also derived R-Squared curves to determine the performance of the proposed DAE-CNN-LSTM model. The closer wind points along linear regression axis shows the better variance and accuracy of the model. All three Offshore Windfarm datasets have obtained an R-Squared correlation of 91.77%, 85.42%, 90.77% respectively. Figs 12–14 illustrate that the data points in Offshore Windfarm 1 are more proportionate to the linear axis compared to Offshore Windfarm 2 and Offshore Windfarm 3. As a result, the R-squared correlation of Offshore Windfarm 1 is higher than other Offshore Windfarm datasets. In general, Figs 12–14 demonstrates the applicability of forecasting measures on offshore wind turbines data collected by NREL.

## Conclusion

A new hybrid approach based on deep auto-encoding and CNN-LSTM neural network is designed to estimate wind power for offshore wind turbine datasets. The DAE model is used as the forecasting engine for the initial outcomes. First, the collected data was screened and those variables were selected that primarily lead to precise predictions. The pre-trained model is then used by CNN-LSTM neural network to forecast actual wind power. Finally, the MAE and RMSE were estimated and their corresponding error ratios were evaluated by comparing them with the widely studied state-of-the-art wind power forecasting models. Experiment findings also proved that the proposed model outperforms alternative approaches in terms effectiveness and sustainability of offshore wind power forecasting.

To enhance forecasting performance in time series datasets, the optimizer function and capabilities of the LSTM neural network can be further improved. For instance, the Gated Neural Network (GRU) is a new and enhanced version of LSTM-based neural networks that can perform faster and train better with minimal data to train forecasting models. In future research, we intend to improve our proposed approaches for short-term wind power forecasting by integrating GRU and other optimization algorithms where limited data is accessible particularly for short-term power forecasting.
